# Bioluminescence Imaging Reveals Dynamics of Beta Cell Loss in the Non-Obese Diabetic (NOD) Mouse Model

**DOI:** 10.1371/journal.pone.0057784

**Published:** 2013-03-06

**Authors:** John Virostko, Armandla Radhika, Greg Poffenberger, Adrienne N. Dula, Daniel J. Moore, Alvin C. Powers

**Affiliations:** 1 Vanderbilt University Institute of Imaging Science, Vanderbilt University, Nashville, Tennessee, United States of America; 2 Department of Medicine, Division of Diabetes, Endocrinology, and Metabolism, Vanderbilt University, Nashville, Tennessee, United States of America; 3 Department of Pathology, Immunology, and Microbiology, Vanderbilt University, Nashville, Tennessee, United States of America; 4 Department of Pediatrics, Vanderbilt University, Nashville, Tennessee, United States of America; 5 Department of Molecular Physiology and Biophysics, Vanderbilt University, Nashville, Tennessee, United States of America; 6 VA Tennessee Valley Healthcare System, Nashville, Tennessee, United States of America; La Jolla Institute for Allergy and Immunology, United States of America

## Abstract

We generated a mouse model (MIP-Luc-VU-NOD) that enables non-invasive bioluminescence imaging (BLI) of beta cell loss during the progression of autoimmune diabetes and determined the relationship between BLI and disease progression. MIP-Luc-VU-NOD mice displayed insulitis and a decline in bioluminescence with age which correlated with beta cell mass, plasma insulin, and pancreatic insulin content. Bioluminescence declined gradually in female MIP-Luc-VU-NOD mice, reaching less than 50% of the initial BLI at 10 weeks of age, whereas hyperglycemia did not ensue until mice were at least 16 weeks old. Mice that did not become diabetic maintained insulin secretion and had less of a decline in bioluminescence than mice that became diabetic. Bioluminescence measurements predicted a decline in beta cell mass prior to the onset of hyperglycemia and tracked beta cell loss. This model should be useful for investigating the fundamental processes underlying autoimmune diabetes and developing new therapies targeting beta cell protection and regeneration.

## Introduction

Type 1 diabetes (T1D) results from the autoimmune destruction of the insulin-producing pancreatic beta cell. The nonobese diabetic (NOD) mouse, the predominant pre-clinical model of type 1 diabetes, is characterized by immune cell infiltration of the pancreatic islet, known as insulitis, followed by beta cell destruction and the development of diabetes [1]. The NOD mouse has proven useful in revealing fundamental autoimmune processes, including the role of T cells [2], autoantibodies [3], and the participation of multiple other immune cell types [4].

A major challenge in the study of T1D in both humans and the NOD mouse is the heterogeneity of disease. The degree of beta cell destruction prior to the onset of hyperglycemia varies among individuals with T1D [5]. Even within the genetically homogeneous NOD mouse population, diabetes has incomplete penetrance, the age of disease incidence varies widely, and environmental factors influence disease pathogenesis [6]. This heterogeneity confounds studies in the NOD mouse, particularly prior to the onset of hyperglycemia, a period thought to be most promising for therapeutic interventions [7]. Given these difficulties, investigators have attempted to identify biomarkers that portend the development of type 1 diabetes [8]. Islet autoimmunity can be identified in the pre-diabetic stage by detection of insulin autoantibodies [9]. However, expression of these markers is transient and does not necessarily predict diabetes progression in the NOD mouse [10]. Indeed, disease progression in the NOD mouse is thought to consist of a multi-stage process characterized by at least two checkpoints. The development of insulitis occurs in all mice, but is thought to be followed by beta cell destruction in only a subpopulation of mice [11]. The incidence and kinetics of this beta cell destruction are not well characterized.

A second challenge in the study of T1D is the inability to measure β cell mass non-invasively and repeatedly in the same mouse or human [12], [13]. A measure of beta cell mass in the NOD mouse would enable monitoring of the progression of T1D and aid the development of therapies. However, assessing the pancreatic beta cell within the intact pancreas is a challenging endeavor. The pancreatic islets, which encompass the beta cell, are small in size, constitute only 1–2% of the total pancreatic mass, and possess no readily identifiable source of imaging contrast. One technique that has proven successful for imaging the native pancreatic beta cell is the transgenic expression of an optical reporter gene under control of a beta cell specific promoter [14]–[17]. We reasoned that combining a mouse model that permits optical imaging of beta cell mass [17] with the NOD mouse would permit noninvasive imaging of beta cell mass during the progression of type 1 diabetes. In this study we employ bioluminescence imaging (BLI) to investigate the relationship between beta cell mass, beta cell function, and the progression of disease in the NOD mouse model.

## Materials and Methods

### Ethics Statement

Animal studies were performed according to guidelines in the *Guide for the Care and Use of Laboratory Animals of the National Institutes of Health*. Animal protocols were approved by the Institutional Animal Care and Use Committee at Vanderbilt University Medical Center (Animal Welfare Assurance Number A3227-01).

### Generation of MIP-Luc-VU-NOD Transgenic Mouse Line

Male MIP-Luc-VU mice [17] were bred with female NOD/ShiLtJ (Jackson Laboratories, Bar Harbor, ME) mice to yield mice expressing luciferase in the pancreatic beta cell on the NOD background. This MIP-Luc-VU-NOD congenic strain was developed utilizing a ‘speed congenic’ process, or marker-assisted breeding strategy, as described by Wong [18]. Briefly, accelerated congenic breeding utilizes genetic analysis of each N2 or higher offspring. PCR primer sets that amplify strain-specific microsatellite loci are used to select the ‘best’ congenic for each successive generation, resulting in a congenic strain in fewer backcross generations. A panel of 61 microsatellite markers, equally spaced throughout the genome (∼30cM intervals), were used to differentiate the genetic background of the originating/donor (MIP-Luc-VU) and target/recipient (NOD/ShiLtJ) mouse strains. Informative markers that distinguish between these strains were selected with the aid of a ‘panel generator’ from http://www.cidr.jhmi.edu/mouse/mmset/html. Using speed congenics, integration of the NOD background was achieved in nine backcrosses and confirmed from chromatogram data using GeneMapper 3.5 software (Applied Biosystems, Foster City, CA). The MIP-Luc-VU-NOD line is being deposited in The Jackson Laboratory Induced Mutant Resource Repository (Bar Harbor, Maine, USA) and can be found by searching the JAX Mice database (http://jaxmice.jax.org/query).

### Bioluminescence Imaging

Bioluminescence imaging (BLI) was performed using an IVIS 200 CCD camera (Xenogen/Caliper, Alameda, CA) as previously described [17], [19], [20]. Anesthesia was induced and maintained with isoflurane (1.5% in 98.5% O2). The substrate D-luciferin (at a concentration of 15 mg/ml in PBS) was injected into the intraperitoneal cavity with a dose of 150 mg/kg. Animals were oriented laterally on the imaging stage, with their left-side facing up toward the camera. To ensure peak bioluminescence was captured, animals were imaged with 1 minute integration times up to 12 minutes post-injection, with peak luminescence typically reached 10 minutes after luciferin administration. Bioluminescence intensity was quantified from the image of peak bioluminescence using Living Image analysis software (Xenogen, Alameda, CA). Equal area regions of interest (ROI) were centered over the bioluminescent region. Photon counting measurements summed bioluminescent intensity for all pixels within the ROI over the integration time. To account for differences in the baseline expression of bioluminescence in individual mice, measurements at each weekly time point were divided by the highest bioluminescence measurement from that mouse to yield normalized bioluminescence values. This highest bioluminescence measurement for each mouse occurred in the first, second, or third week of imaging (∼8 weeks of age), prior to the onset of beta cell loss.

### Immunocytochemistry

Dissected mouse pancreata were fixed using methods previously described [21]. Pancreata were then equilibrated in increasing concentrations of ethanol, embedded in hot paraffin, and sectioned. Sections were dewaxed and rehydrated with ethanol, washed with PBS, blocked with normal donkey serum, and incubated with primary antibodies overnight at 4°C. Guinea pig anti-insulin IgG (1∶1000) was obtained from Dako North America, Inc. (Carpinteria, CA), goat anti-luciferase (1∶500) was from Promega (Madison, WI), and rat anti-CD45R/B220 (1∶500) was from BD Biosciences (San Jose, CA). Secondary antibodies conjugated with Cy2 or Cy3 fluorophores (1∶500, Jackson ImmunoResearch Laboratories, Inc., West Grove, PA) were applied to the tissue sections for 1 hr at room temperature. For hematoxylin and eosin staining, slides were immersed in hematoxylin for 3 minutes, washed in distilled water and acid ethanol, immersed in eosin for 1 minute, and placed in serial concentrations of ethanol prior to mounting. The luciferase and insulin staining shown in Figure S2 was performed on cryosections, as previously described [22].

To determine beta cell mass in the pancreas of MIP-Luc-VU-NOD mice, pancreatic sections spaced by 250 µm from three different levels of the pancreatic tissue block were immunolabeled for insulin, as described above. Following overnight incubation with insulin primary antibody, slides were incubated for 2 hours in 1∶200 HRP-conjugated donkey anti-guinea pig antibody. DAB substrate (VectorLabs, Burlingame, CA) was applied for 1 minute, washed with distilled water, and counterstained for eosin. Pancreatic beta cell area measurements were performed using a ScanScopeCS brightfield slide scanner and ImageScope software (Aperio Technologies, Inc., Vista, CA). ImageScope software was used to calculate the insulin- and eosin-positive areas of each cross-section, and the pancreatic beta cell area was defined as (insulin area)/(insulin area plus eosin area). The beta cell mass was then determined by multiplying the relative beta cell area by the total pancreatic weight.

Insulitis was determined from H&E stained pancreatic sections [23]. Islets were marked and given scores according to the following scale: S0– no evidence of islet infiltration, S1– islet infiltration confined to perimeter, S2– islet infiltration <50% of islet area, S3– islet infiltration >50% of islet area, S4– islet covered by infiltrate. Islets representative of each score are shown in Figure S1.

### Blood Glucose Measurement and In Vivo Insulin Secretion

Blood glucose measurements were obtained using tail vein blood measured with an Accu-chek glucose meter (Roche Diagnostics, Indianapolis, IN). Hyperglycemia was defined as blood glucose above 200 mg/dl. For glucose tolerance testing, baseline blood glucose was measured after a six hour fast and then mice were challenged with an I.P. injection of 2 g/kg of glucose followed by blood glucose measurement at 15, 30, 60, 90, and 120 minute increments. For insulin secretion, mice were challenged with an I.P. injection of 2 g/kg glucose/arginine after a six hour fast. Retro-orbital eye bleeds were performed prior to injection and at 15 minutes after injection. Samples were centrifuged at 4°C for 15 minutes and clear supernatants were collected for insulin measurement by radioimmunoassay [21].

### Insulin Content

Pancreata were excised and cleaned of connective tissue, blotted dry, and weighed. Tissue was homogenized in acid alcohol (1 ml 12 M HCL/110 mL 95% ethanol) and incubated for 48 h at 4°C under mild agitation, as described [21]. The homogenate was centrifuged at 2,500 rpm for 30 min at 4°C. The supernatant was collected for radioimmunoassay and stored at −20°C [21].

### Statistical Analysis

Data are expressed as means ± standard error of the mean (SEM). An unpaired, two-tailed t test was used to compare experimental results between two groups. For experiments with more than two groups, ANOVA was used with post-hoc Tukey’s honest significance test or test for linear trend, as indicated (Prism, Graphpad, La Jolla, CA). For predictive modeling, a binary logistic regression analysis was performed using SPSS software (IBM, Armonk NY).

## Results

### MIP-Luc-VU-NOD Display Characteristics Similar to NOD Mice

MIP-Luc-VU-NOD mice developed the insulitis characteristic of the NOD mouse model. For example, an islet from a representative 22 week-old female MIP-Luc-VU-NOD mouse displayed lymphocyte infiltration of the islet by both H&E staining (Figure S1A) and immunofluorescence staining for the B220 pan B lymphocyte marker (Figure S1B). Insulitis scoring of islets from female MIP-Luc-VU-NOD mice at 8, 12, and 22 weeks of age displayed a progression toward increased insulitis (Figure S1C). Mice at 8 weeks of age displayed little infiltration, while mice at 22 weeks of age showed infiltration in every islet examined. Islets representative of each insulitis score are shown in Figure S1D. Luciferase expression was confined to beta cells (Figure S2), was similar across several islets and mice, and was comparable to results found with MIP-Luc-VU mice [17]. As found with MIP-Luc-VU mice, a population of beta cells expressed luciferase with similar expression among all mice analyzed. Thus bioluminescence imaging reflects the signal from a population of beta cells, rather than every beta cell. In the MIP-Luc-VU mouse, these islets have normal morphology and function [17]. Weekly blood glucose measurements demonstrated the progression toward hyperglycemia in both female and male MIP-Luc-VU-NOD mice. These results suggest that MIP-Luc-VU-NOD mice develop autoimmune diabetes similar to previously published studies of NOD mice [1], [6] and express luciferase similar to MIP-Luc-VU mice [17].

### Bioluminescence Intensity Decreases Temporally in MIP-Luc-VU-NOD Mice

**Figure 1:**
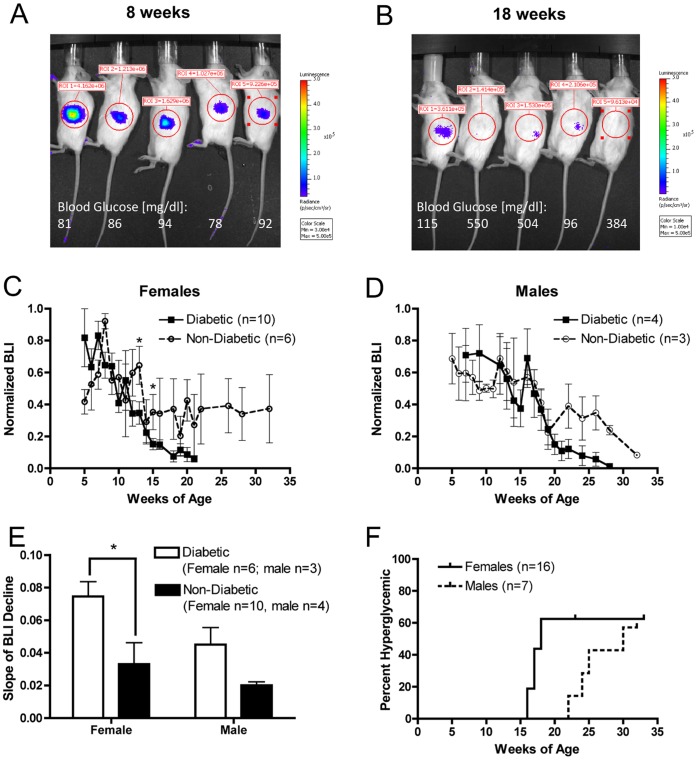
MIP-Luc-VU-NOD mice display a decline in bioluminescence with increasing age. A) A representative bioluminescence image from five female MIP-Luc-VU-NOD mice at 8 weeks of age, with blood glucose shown below each mouse. B) Bioluminescence image from the same five mice at 18 weeks of age reveals a decline in bioluminescence and rise in random blood glucose in some mice. C) Quantification of bioluminescence from female MIP-Luc-VU-NOD mice reveals a greater decline in BLI in mice that ultimately became diabetic, but also a decline in BLI of mice that did not become diabetic over the course of the study. (*P<0.05, diabetic versus non-diabetic at week indicated). D) Male MIP-Luc-VU-NOD mice also display reduced BLI with increasing age in diabetic and non-diabetic mice, however BLI persists longer than in female mice. E) The slope of the temporal decline in BLI is greater in mice that become diabetic. (*P<0.05). F) Kaplan Meier estimator displays the incidence of hyperglycemia (blood glucose >200 mg/dl) in male and female mice, with earlier incidence in female mice.

Repeated bioluminescence imaging of female MIP-Luc-VU-NOD revealed a decline in bioluminescence intensity as mice aged. Weekly BLI, initiated when mice were between five and seven weeks old, was maximum in the first, second, or third week of measurement and then declined in every MIP-Luc-VU-NOD mouse imaged (Figure 1A, 1B). In contrast, we have previously shown that bioluminescence imaging of beta cell mass is stable over a 6 month period in MIP-Luc-VU mice on an FVB background [17]. The blood glucose of MIP-Luc-VU-NOD mice increased in some animals by 18 weeks of age (Figure 1A, 1B). Bioluminescence intensity decreased temporally in all female MIP-Luc-VU-NOD mice, but animals that ultimately became diabetic at later time points displayed a greater decrease in BLI beginning in week 13 (Figure 1C). Male MIP-Luc-VU-NOD mice also displayed a decrease in BLI, with mice that ultimately became diabetic at later time points also displaying a greater decrease in BLI (Figure 1D). In contrast with female mice, the decline in bioluminescence intensity was slower in male mice. The slope of decline in BLI was greater in mice that became diabetic (Figure 1E). The incidence of diabetes occurred at an earlier time point in female MIP-Luc-VU-NOD mice than male mice (Figure 1F).

### Bioluminescence Intensity Reflects Other Metrics of Beta Cell Mass

**Figure 2:**
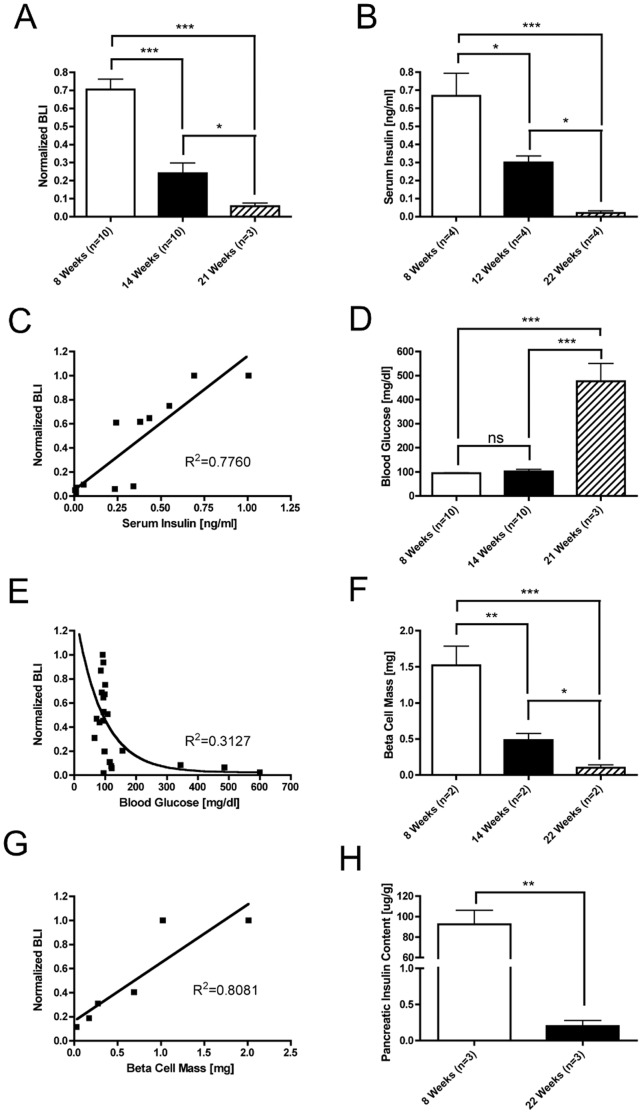
Temporal decrease in bioluminescence of female MIP-Luc-VU-NOD mice corresponds with measures of beta cell mass. A) Normalized bioluminescence displays a decline in bioluminescence with increasing age. B) Serum insulin measurements decrease with increasing mouse age. C) Normalized bioluminescence correlates with serum insulin (same mice from Panel B). D) Blood glucose is normal at 8 and 14 weeks of age but mice become hyperglycemic by 21 weeks of age. E) Blood glucose measurements display an inverse exponential relationship with normalized BLI, as blood glucose is unaltered with initial declines in BLI (same mice from Panel D). F) Morphometric measurements of beta cell mass reveal a decrease in beta cell mass with increasing age. G) Normalized bioluminescence correlates with post-mortem morphometric measurement of beta cell (same mice from Panel F). H) Pancreatic insulin content measurements decrease with mouse age. Each panel displays only female mice. (All panels: *P<0.05, **P<0.01, ***P<0.001, post-hoc Tukey’s honest significance test).

In order to determine whether bioluminescence correlates with measures of beta cell mass, BLI, serum insulin measurements, blood glucose, morphometric measurement of insulin immunostaining, and pancreatic insulin content measurements were performed at serial time points in female MIP-Luc-VU-NOD mice. Mice were randomly selected for these assays from a cohort of 20 mice. Normalized bioluminescence intensity decreased with increasing mouse age (Figure 2A). Serum insulin measurements similarly decreased with mouse age (Figure 2B) and correlated with bioluminescence in individual mice (Figure 2C). All mice were normoglycemic at 14 weeks of age but some became hyperglycemic at 21 weeks of age (Figure 2D). Normoglycemia was preserved despite initial declines in BLI and hyperglycemia ensued only after a large decline in BLI (Figure 2E). Morphometric measurement of beta cell mass declined with increasing age in MIP-Luc-VU-NOD mice (Figure 2F) and this correlated with normalized bioluminescence measurements (Figure 2G). Furthermore, pancreatic insulin content measurements also decreased with increasing mouse age (Figure 2H).

### Bioluminescence Imaging Reflects Disease Heterogeneity

**Figure 3:**
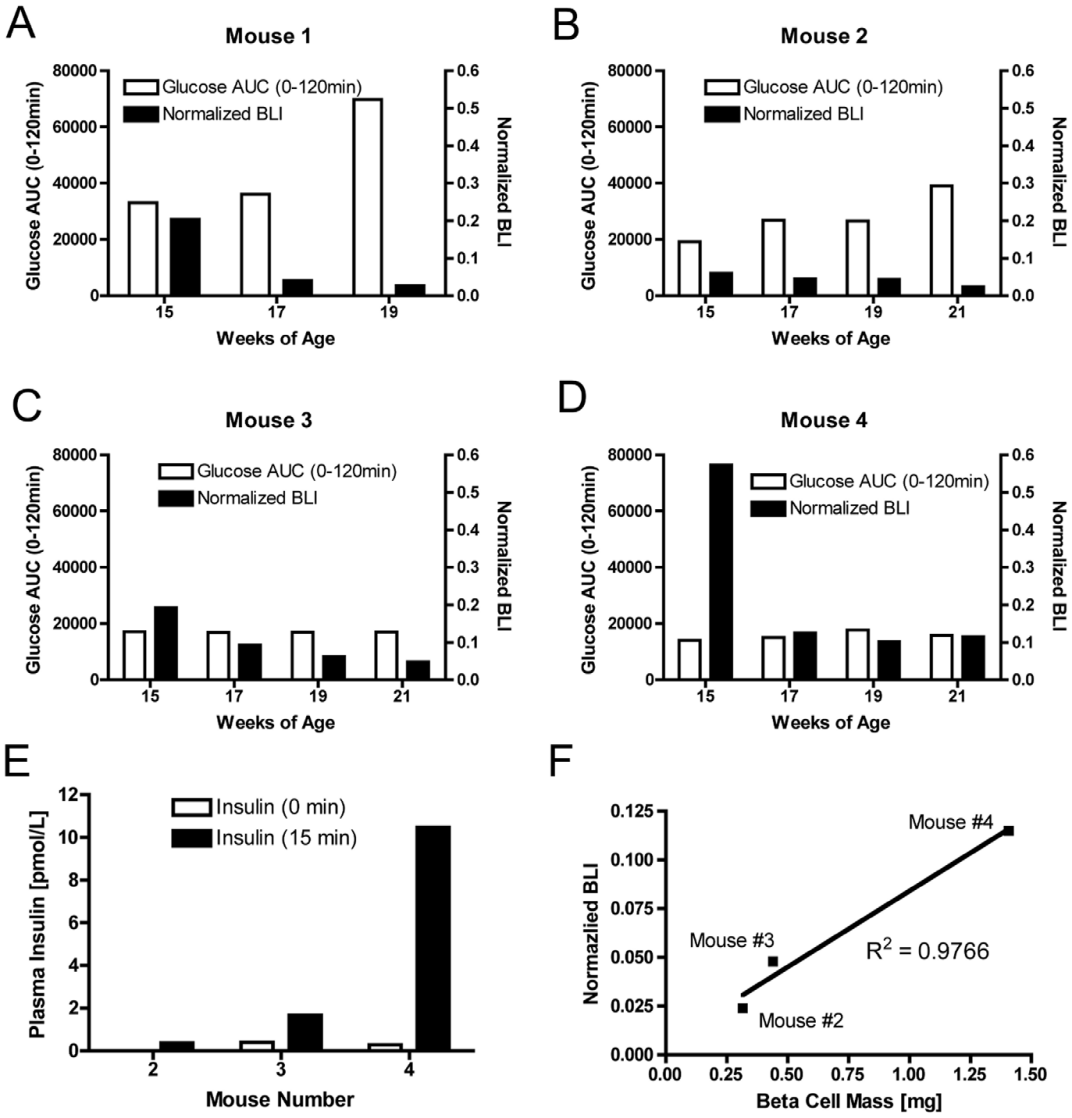
Heterogeneity of diabetes incidence in female MIP-Luc-VU-NOD mice is reflected by BLI. Glucose tolerance tests and bioluminescence imaging were performed on four female MIP-Luc-VU-NOD mice at 15, 17, 19, and 21 weeks of age. Normalized bioluminescence measurements varied inversely with area under the curve during glucose tolerance testing in A) mouse #1 and B) mouse #2. In C) mouse #3 and D) mouse #4 bioluminescence intensity decreased but glucose tolerance testing continued to be normal. Note that mouse #1 died prior to week 21. E) Glucose/arginine-stimulated insulin secretion revealed impaired insulin secretion in mouse #2 and mouse #3. F) Normalized bioluminescence at 21 weeks of age correlates with post-mortem morphometric measurement of beta cell mass.

Since the NOD mouse model displays heterogeneity in the occurrence, severity, and age of onset of diabetes, four female MIP-Luc-VU-NOD mice were followed with biweekly BLI and glucose tolerance testing at 15, 17, 19, and 21 weeks of age. Glucose tolerance testing (GTT) revealed variability in the progression toward diabetes in these mice (Figure S3). BLI likewise reflected heterogeneous progression of diabetes in these same mice (Figures 3A–D). For example, mouse #1 had an abnormal GTT response at 15 weeks of age and increasingly blunted glucose response and a decline in BLI (Figure 3A). Mouse #2 had an abnormal GTT at 17 weeks of age and increasingly abnormal glucose response up to 21 weeks of age along with a decline in BLI (Figure 3B). In contrast, mouse #3 (Figure 3C) and mouse #4 (Figure 3D) had normal glucose tolerance testing despite a decline in BLI. Of note, the BLI decline in mouse #4 was not as great as in the other three mice, and did not exceed a 90% decline in BLI. Glucose/arginine stimulation of insulin secretion in these same mice revealed impaired insulin secretion in mouse #2 and mouse #3 at 21 weeks of age (Figure 3E). Post mortem morphometric measurement of beta cell mass in the three surviving animals revealed a direct, linear correlation between the normalized BLI measurement and beta cell mass (Figure 3F). These results indicate that BLI provides a measure of beta cell mass that does not necessarily correlate with functional glucose tolerance testing during the progression of diabetes.

### Decline in Bioluminescence Intensity Precedes Hyperglycemia

**Figure 4:**
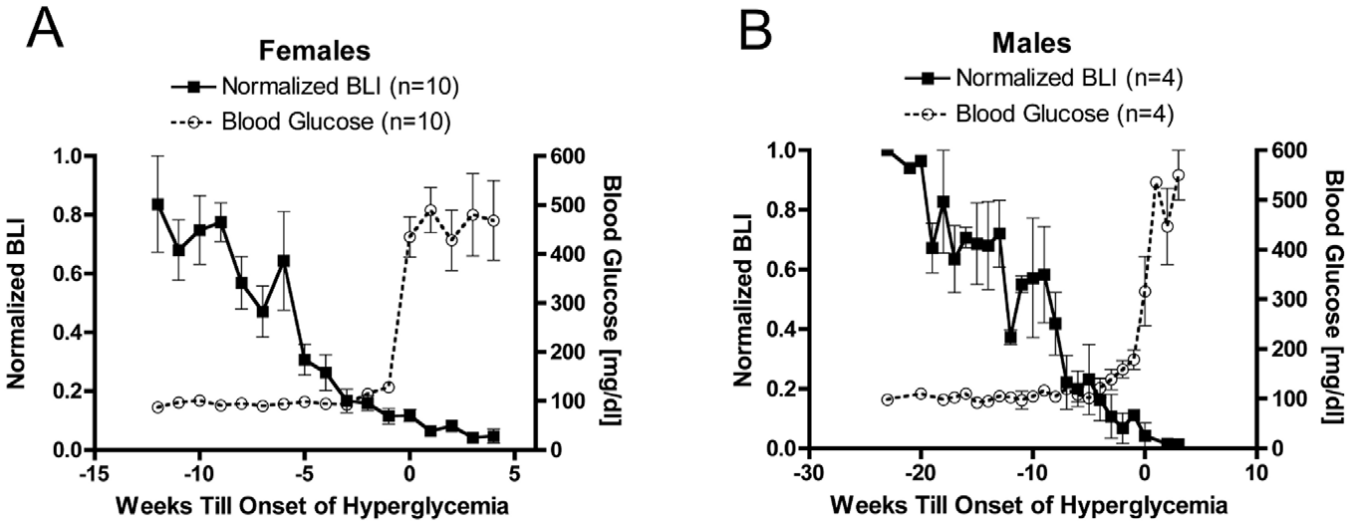
Decline in bioluminescence in MIP-Luc-VU-NOD mice precedes the onset of hyperglycemia. A) Female mice that become diabetic (blood glucose >200 mg/dl) have a gradual reduction in BLI with increasing age that precedes hyperglycemia by four weeks. (P<0.001 ANOVA and post-hoc test for linear trend, BLI and blood glucose). B) Male mice that become diabetic also display a reduction in BLI preceding hyperglycemia, with a slower decline in BLI and later onset of diabetes. (P<0.001 ANOVA and post-hoc test for linear trend, BLI and blood glucose).

Blood glucose measurements of female MIP-Luc-VU-NOD mice that became diabetic were initially normoglycemic despite a gradual decline in bioluminescence over the period of several weeks prior to the onset of hyperglycemia (Figure 4A). This is similar to human T1D where diabetes does not occur until most beta cell mass is lost [24]. Diabetes ensued only after bioluminescence intensity had declined to 9.9±2.0% of the initial bioluminescence value, on average. Bioluminescence intensity continued to decrease in MIP-Luc-VU-NOD mice after the onset of hyperglycemia (Figure 4A). Female MIP-Luc-VU-NOD mice that did not become hyperglycemic by 32 weeks of age displayed an initial decline in BLI that stabilized at approximately 40% of the maximum BLI (Figure S4A). These mice maintained insulin secretion at 32 weeks of age (Figure S4B) and beta cell mass (1.28+/−0.48 mg).

Male MIP-Luc-VU-NOD that became diabetic also demonstrated a decline in bioluminescence intensity prior to the onset of hyperglycemia (Figure 4B). The onset of hyperglycemia was later in male mice compared with female mice. Additionally, the onset of hyperglycemia in male mice occurred after a larger decline in bioluminescence intensity (1.7±0.2% of the initial BLI) than in female mice. These results indicate that BLI reflects a gradual decline in beta cell mass that reaches a critical threshold prior to the onset of hyperglycemia.

### Predictive Modeling

A binary logistic regression analysis was conducted to predict the incidence of diabetes in female MIP-Luc-VU-NOD mice using bioluminescence intensity as a predictor. Bioluminescence intensity at three weeks prior to the onset of diabetes was a reliable predictor for distinguishing mice that became diabetic from mice that did not become diabetic. The odds ratio of developing diabetes was small given the limited sample size, but significant (p = 0.024).

A predictive binary logistic model based upon the bioluminescence value at three weeks prior to diabetes incidence was constructed using the following equation:
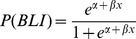
with β = −9.3231 and α = 4.072 and the dependent variable as bioluminescence value at three weeks prior to diabetes onset. This developed model had a sensitivity of 0.7 and specificity of 0.9. Prediction of the incidence of diabetes from bioluminescence values at 3 weeks prior to the event was successful for seven of the ten mice that developed diabetes.

## Discussion

We report the development of a new mouse model in which autoimmune diabetes can be non-invasively predicted by bioluminescence imaging (BLI). Building upon our previous experience using bioluminescence imaging to measure beta cell mass [16], [17], we bred MIP-Luc-VU mice, which express a luciferase optical reporter gene in the beta cell, with the NOD mouse, a widely used model of type 1 diabetes. These mice, known as MIP-Luc-VU-NOD, developed hyperglycemia progressively with increasing age, with a higher incidence and earlier age of onset in female mice compared with male mice, as previously demonstrated in other NOD colonies [6]. The MIP-Luc-VU-NOD model preserves the insulitis phenotype of the NOD mouse as demonstrated by insulitis scoring of MIP-Luc-VU-NOD islets, with increased islet infiltration as animals age. Longitudinal bioluminescence imaging in this mouse model demonstrated a decline in bioluminescence that preceded the onset of diabetes. A predictive model built from this imaging data indicates that BLI can be used to predict the incidence of diabetes in MIP-Luc-VU-NOD mice three weeks prior to the onset of hyperglycemia. These results demonstrate that it is possible to noninvasively image beta cell mass in a model of type 1 diabetes and that BLI reflects beta cell destruction prior to the onset of hyperglycemia.

Longitudinal studies of bioluminescence intensity and blood glucose in the same MIP-Luc-VU-NOD mice reveal that bioluminescence decreases weeks before the onset of hyperglycemia. Indeed, at the onset of hyperglycemia the bioluminescence intensity has decreased by an average of 90%. A drastic reduction in beta cell mass prior to the onset of hyperglycemia has previously been noted in other rodent models [25]. Bioluminescence imaging in this mouse model can thus be used to monitor autoimmune loss of beta cell mass during the period in which mice are normoglycemic. Glucose tolerance testing of mice and comparison with bioluminescence imaging indicates that bioluminescence measurement can detect a decrease in beta cell mass prior to abnormal glucose tolerance testing, as well. Bioluminescence accurately reflected beta cell mass and identified a mouse with reduced beta cell mass which had not yet displayed abnormal glucose tolerance (mouse #3, Fig. 3). Glucose/arginine stimulation of insulin secretion correlated with bioluminescence measurements in these animals, supporting its role as an early biomarker of beta cell dysfunction [8].

The decline in bioluminescence in MIP-Luc-VU-NOD mice correlates with morphometric measurements of beta cell mass, plasma insulin, and pancreatic insulin content. A caveat of bioluminescence imaging is that the spatial resolution is incapable of resolving individual islets; instead the BLI signal reflects the sum of all beta cell bioluminescence. Bioluminescence measurements of this new model reflect beta cell mass during the progression of the NOD diabetic phenotype. This contrasts with a previous report using an inducible activator of luciferase to monitor the NOD mouse, which found that the decline in bioluminescence outstripped the decline in beta cell mass [26]. The use of an inducible bioluminescence protein in this previous report may partially explain this discrepancy, as bioluminescence expression in the MIP-Luc-VU-NOD mouse is persistent, rather than inducible. Indeed the decline in bioluminescence demonstrated in this study was more gradual than the decline in the inducible model. This indicates that the speculation that inflammation lowers BLI in the inducible model [26] is not a factor in the MIP-Luc-VU-NOD model. Furthermore, the current study utilized the NOD/ShiLtJ model and displayed a larger decline in beta cell mass that is more representative of the NOD phenotype than the NOD-LtJ strain used in the inducible study [26]. Of note, different strains of NOD mice have demonstrated different degrees of disease penetrance [27].

In the sample of mice imaged, the incidence of spontaneous diabetes in female MIP-Luc-VU-NOD agrees with the 60% to 80% incidence reported for female mice while the incidence in male mice was higher than the figure of 20% to 30% reported for males in the small sample examined [6]. The incidence of diabetes has been shown to differ between different strains [27]. Furthermore, environmental factors [28], diet [29], and exposure to pathogens [30] alter the incidence of diabetes in NOD mice. In contrast to previous theories that beta cell destruction occurred only in a subset of NOD mice [11], this study indicates that beta cell loss occurs in all NOD mice, as assessed by bioluminescence imaging. However, mice that became hyperglycemic lost over 90% of their BLI, while mice that remained normoglycemic during the study period maintained approximately 40% of their initial bioluminescence intensity, although there was a significant amount of variation in this cohort. This variation may result in part from the heterogeneity of disease penetrance in NOD mice.

The monitoring of type 1 diabetes developed in the MIP-Luc-VU-NOD model should allow for studies that increase our understanding of the NOD model as well as testing new interventions and monitoring disease. The combination of bioluminescence measurements of beta cell mass with longitudinal monitoring of insulitis [31] may yield insight into the relationship between beta cell infiltration and death. Several therapies developed in the NOD strain, such as anti-CD3 [32] are now being tested as clinical investigations in humans with type 1 diabetes [33]. Since numerous interventions have proven effective in the pre-diabetic stage in the NOD model, BLI should be useful in testing new treatments prior to functional impairment [34]. Bioluminescence imaging can be used as an early reporter of beta cell loss and help identify important therapeutic windows. Furthermore, by measuring beta cell mass, the timing and dosage of treatment can be standardized to the status of beta cell loss and the therapeutic response can be monitored.

## Supporting Information

Figure S1 **MIP-Luc-VU-NOD mice display increasing insulitis with age.** A) H&E stain shows lymphocyte infiltration of the pancreatic islet. B) Immunofluorescence staining for insulin (green) and the CD45R/B220 pan B lymphocyte marker (red) reveal lymphocyte infiltration and beta cell loss in MIP-Luc-VU-NOD islets. C) Insulitis scoring from H&E sections of MIP-Luc-VU-NOD mice at 8, 12, and 22 weeks of age displays increased islet infiltration with mouse age (S0 no infiltration; S4 severe infiltration). Sections from 3 mice at each age were scored for insulitis. D) Representative islet of each score outlined with a blue dashed line.
(TIF)

Figure S2 **Immunocytochemistry for insulin (green) and luciferase (red) shows staining of beta cells for luciferase.**
(TIF)

Figure S3 **Heterogeneity of diabetes incidence in female MIP-Luc-VU-NOD mice is reflected by glucose tolerance testing.** Glucose tolerance tests was performed on four female MIP-Luc-VU-NOD mice at A) 15, B) 17, C) 19, and D) 21 weeks of age. Note that mouse #1 died prior to week 21.
(TIF)

Figure S4 **Female MIP-Luc-VU-NOD mice that did not become hyperglycemic by 32 weeks of age were followed with weekly bioluminescence and blood glucose measurements.** A) Bioluminescence intensity declined but then stabilized at approximately 40% of the maximum BLI. Blood glucose measurements remained normal. B) Glucose and arginine-stimulated insulin secretion revealed the maintenance of insulin secretion in these animals.
(TIF)
