# Supplementary figures and images for: Bioluminescence Imaging Reveals Dynamics of Beta Cell Loss in the Non-Obese Diabetic (NOD) Mouse Model
Source: PLoS One. 2013 Mar 6;8(3):e57784. doi: 10.1371/journal.pone.0057784 (PMC3590285; doi:10.1371/journal.pone.0057784)

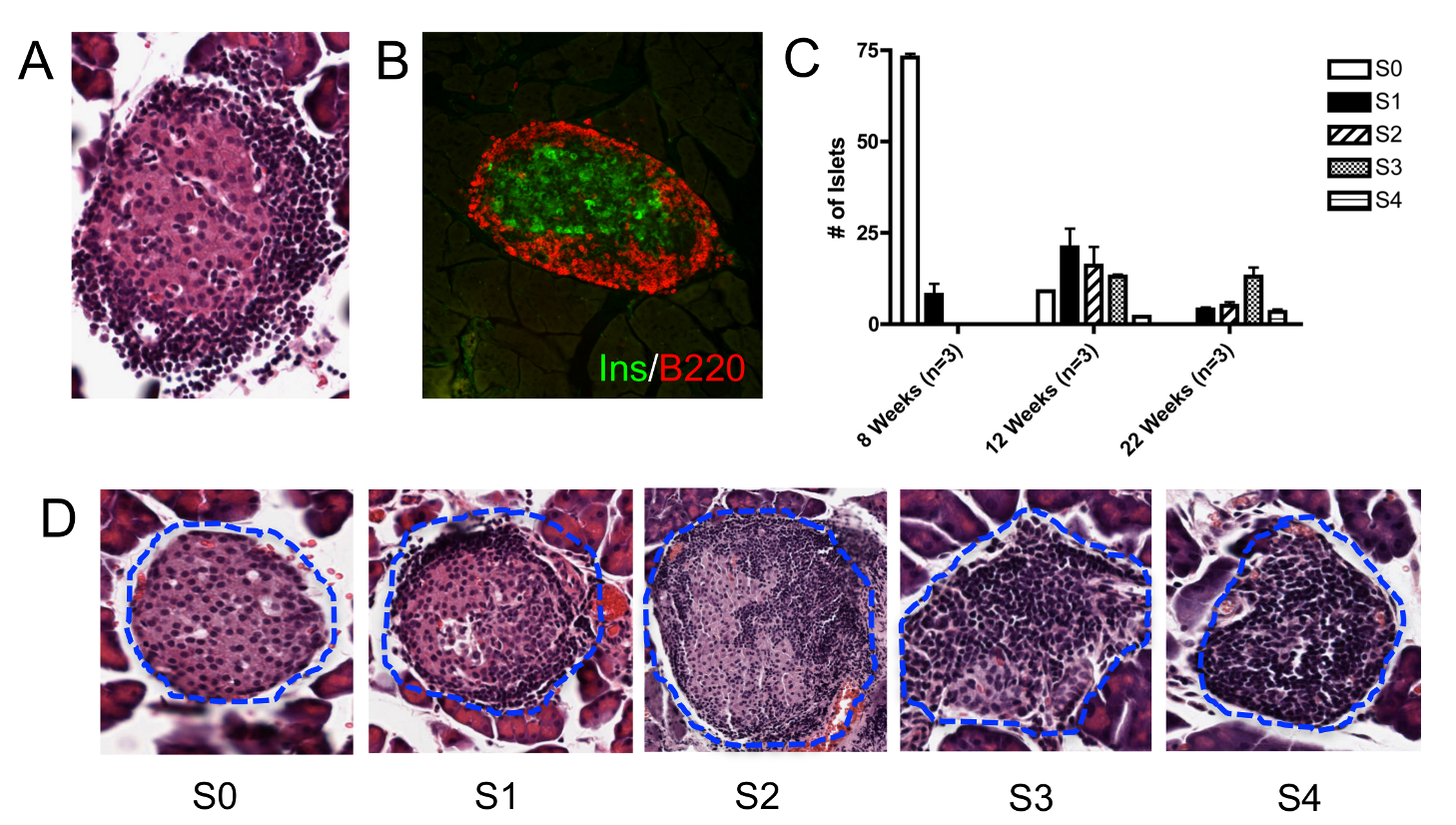

Supplement: Figure S1 — MIP-Luc-VU-NOD mice display increasing insulitis with age. A) H&E stain shows lymphocyte infiltration of the pancreatic islet. B) Immunofluorescence staining for insulin (green) and the CD45R/B220 pan B lymphocyte marker (red) reveal lymphocyte infiltration and beta cell loss in MIP-Luc-VU-NOD islets. C) Insulitis scoring from H&E sections of MIP-Luc-VU-NOD mice at 8, 12, and 22 weeks of age displays increased islet infiltration with mouse age (S0 no infiltration; S4 severe infiltration). Sections from 3 mice at each age were scored for insulitis. D) Representative islet of each score outlined with a blue dashed line. (TIF) [file pone.0057784.s001.tif]

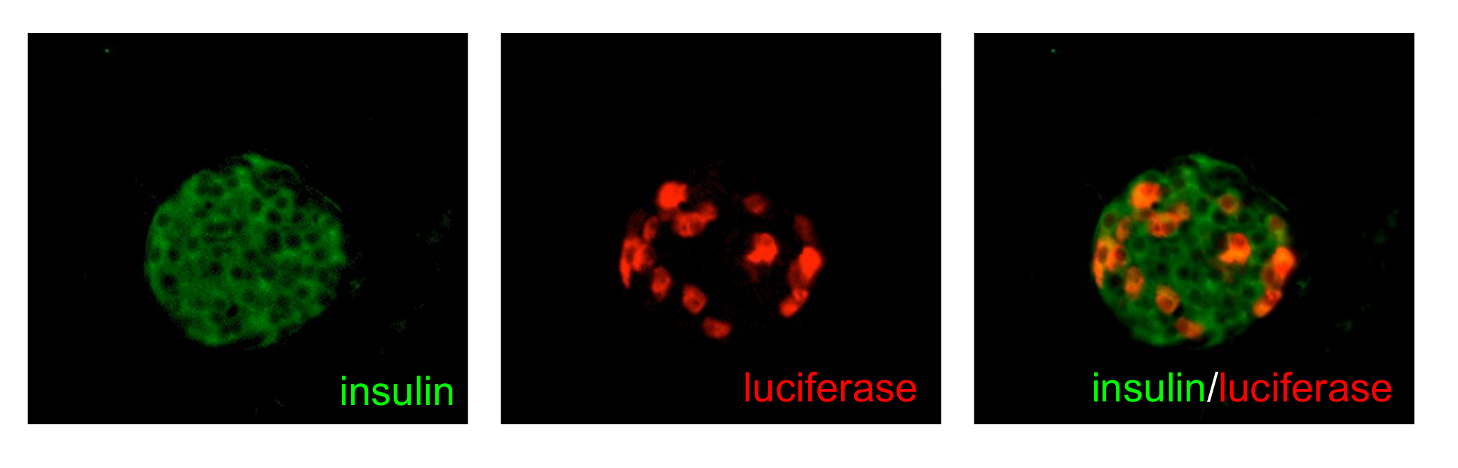

Supplement: Figure S2 — Immunocytochemistry for insulin (green) and luciferase (red) shows staining of beta cells for luciferase. (TIF) [file pone.0057784.s002.tif]

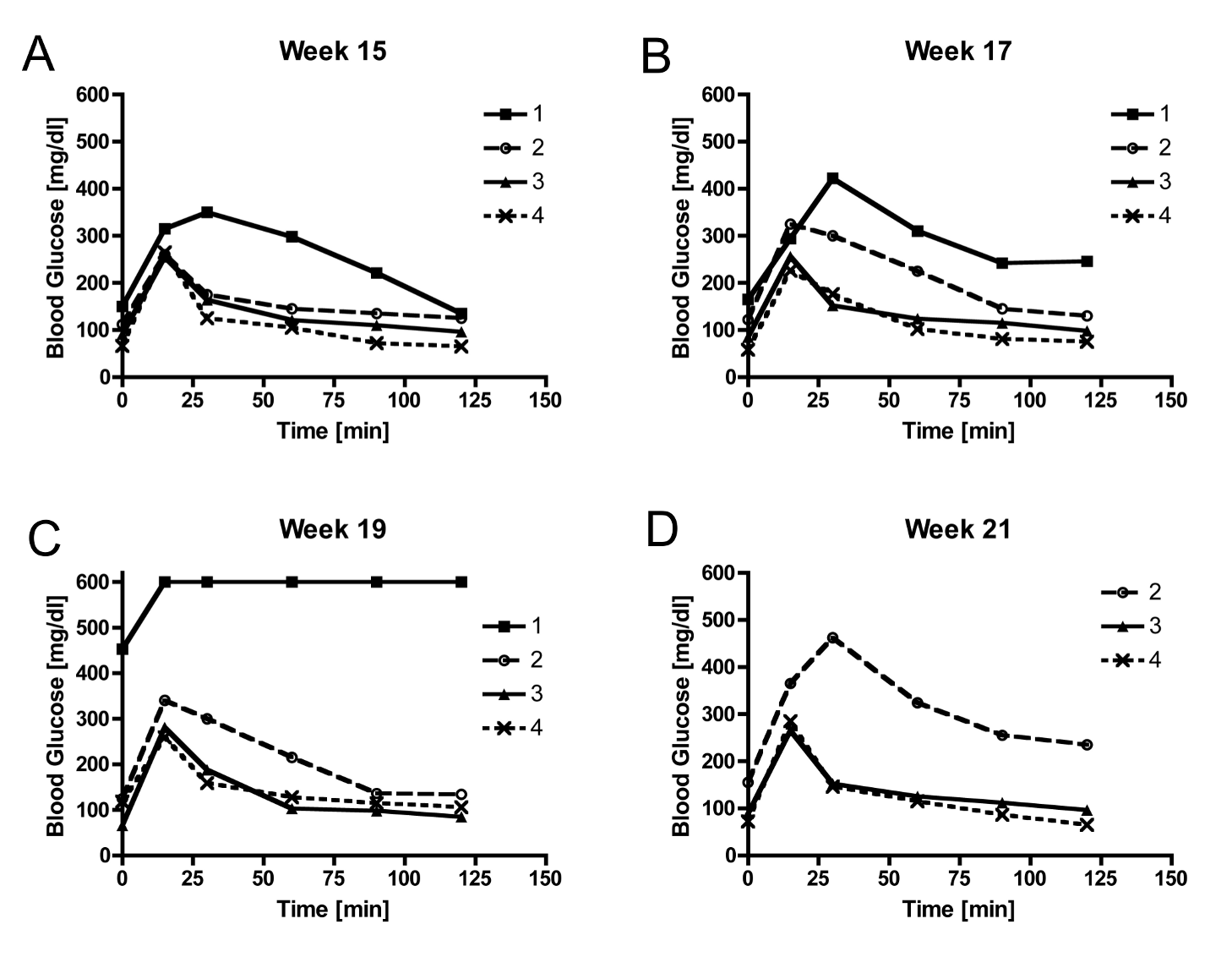

Supplement: Figure S3 — Heterogeneity of diabetes incidence in female MIP-Luc-VU-NOD mice is reflected by glucose tolerance testing. Glucose tolerance tests was performed on four female MIP-Luc-VU-NOD mice at A) 15, B) 17, C) 19, and D) 21 weeks of age. Note that mouse #1 died prior to week 21. (TIF) [file pone.0057784.s003.tif]

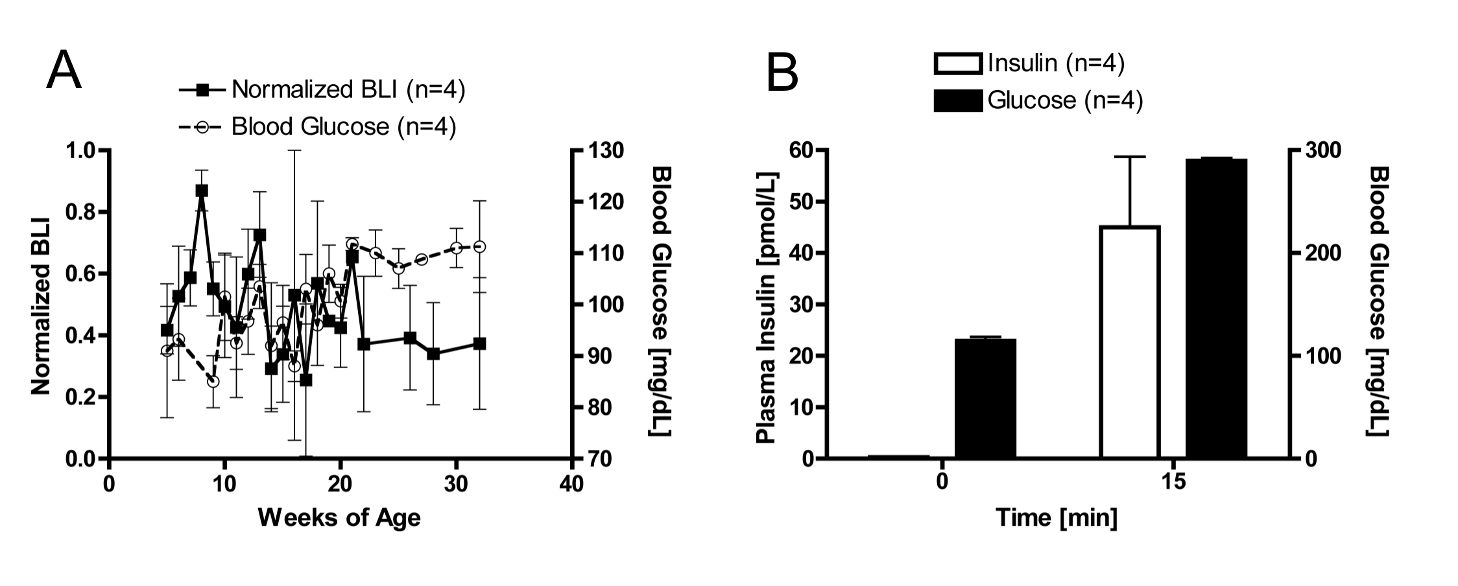

Supplement: Figure S4 — Female MIP-Luc-VU-NOD mice that did not become hyperglycemic by 32 weeks of age were followed with weekly bioluminescence and blood glucose measurements. A) Bioluminescence intensity declined but then stabilized at approximately 40% of the maximum BLI. Blood glucose measurements remained normal. B) Glucose and arginine-stimulated insulin secretion revealed the maintenance of insulin secretion in these animals. (TIF) [file pone.0057784.s004.tif]
